# Methylated DNA as Promising Marker for Early Diagnosis of Cancer

**DOI:** 10.4172/1948-593X.1000e108

**Published:** 2012-10-29

**Authors:** Hafiz Ahmed

**Affiliations:** Department of Biochemistry and Molecular Biology, University of Maryland School of Medicine & Greenebaum Cancer Center Baltimore, USA

Cancer is the leading cause of death worldwide estimating 12.7 million new cancer diagnoses and 7.6 million deaths in the year 2012 [1]. It is estimated that new cancer diagnoses will double by 2020 and nearly triple by 2030 [2]. However, cancer death can be reduced or prevented if detected at their early stages. Conventional histopathology, immunohistochemistry or image based screening tools such as mammography for breast cancer and chest X-ray for lung cancer may render specific detection of cancer, but may not be sensitive enough for early detection of the disease. Moreover, some of these tools are invasive and therefore, it is imperative to develop noninvasive techniques that distinguish between patients with and without cancer, as well as between stages of cancer. The introduction of advanced sophisticated technologies like proteomics [3], mass spectrometry, microarray (mRNA, micro RNA [4], protein, lectin, glycan [5]), automated DNA sequencing, comparative genomic hybridization, and epigenetics (DNA methylation) have allowed to search for new cancer biomarkers that may be useful for non-invasive (or minimally invasive) early detection from biological fluids such as serum, urine, sputum as well as fluid-derived exosomes [6] and circulating tumor cells [7] and thereby prevention of the disease.

Proteomics allows both qualitative and quantitative assessment of changes in protein expression related to specific cellular responses [3]. Many protein markers for various cancers such as NMP22 (bladder cancer), CEA (colorectal cancer), CA15-3 and Her2/Neu (breast cancer), alpha-fetoprotein (liver cancer), CA-19-9 (pancreatic cancer), and CA-125 (ovarian cancer) have been identified using proteomics [3]. The alteration in protein glycosylation on the cell surface and in body fluids such as serum has been shown to correlate with the progression of cancer. Thus, a high-throughput technique for quantitative analysis of glycan structure on glycoproteins may be useful for identifying new glycoprotein biomarkers suitable for early cancer detection. The fucosylated haptoglobin has recently been identified as a marker for pancreatic cancer using this approach [5].

Gene expression profiling or microarray analysis has been now a standard technique which enabled the measurement of thousands of genes in a single RNA sample [8]. Although a variety of microarray platforms have been developed over the years to accomplish this, but the basic principle is that a glass slide or membrane is spotted or “arrayed” with DNA fragments or oligonucleotides that represent specific gene coding regions. Fluorescently labeled (usually Cy3 or Cy5) purified RNA from tumor specimen is then hybridized to the oligonucleotide array. APRIL/TNFSF13 (a TNF superfamily ligand) has recently been identified as a novel clinical chemo-resistance biomarker in colorectal adenocarcinoma by this approach [9]. Moreover, microRNAs (miRNAs) are emerging as promising biomarkers for cancer detection because their expression is frequently dysregulated in cancer and their expression patterns in human cancer appear to be tissue-specific. The miRNA *miR-141* has been identified as a marker for prostate cancer [7]. Comparative genomic hybridization allows detection of chromosomal gains and losses in genomic complement [10] and several diagnostic molecular markers such as K ras mutation (colon cancer) [11] and p53 mutation (bladder cancer, head and neck cancer) [12] have been identified by this method. A recently described gene fusion between TMPRSS2 and ETS family genes in prostate cancer may have clinical applications in diagnosis, prognosis and therapy [13].

The detection of circulating tumor cells (CTCs) may represent an early indication of micro-metastasis or aggressive tumors which are able to shed tumor cells into the blood [14]. The CTCs are captured using antibody labeled magnetic beads and characterized for gene expression analysis by RT-PCR. The detection of CTCs is being used as a prognostic test in patients with metastatic cancers of the breast, prostate and colon. The exacerbated release of 40-100 nm membrane vesicles (called exosomes) in tumor cells, suggests an important role of exosomes in diagnosis and biomarker studies [15]. They are found *in vivo* in body fluids such as blood, urine, amniotic fluid, malignant ascites, bronchoalveolar lavage fluid, synovial fluid, and breast milk. Prostate cancer derived urine exosomes are shown to contain two known prostate cancer biomarkers, PCA-3 and TMPRSS2: ERG, showing the potential for diagnosis and monitoring cancer patient’s status [16].

Epigenetic alterations, including hypermethylation of CpG islands in the gene promoters are believed to be early events in neoplastic progression [17]. Hypermethylation of tumor suppressor gene promoters contributes to their silencing during the neoplastic process (Figure 1). Thus, methylated gene promoters can serve as markers for the detection of cancer from clinical specimens such as tissue biopsies or body fluids [18]. For example, GSTP1, RASSF1A, RAR2 and LGALS3 promoters are frequently methylated [18,19] in prostate cancer; while ARF, APC, and DAPK have been found methylated in bladder cancer [18]. DNA is stable and its modifications can be reliably detected both qualitatively and quantitatively by PCR-based techniques. PCR also allows detection of as few as one cancer cell (or genome copy) in a background of thousands of normal cells, thereby permitting detection of a cancer before it can be visualized by imaging or traditional pathology. Therefore, methylated DNA sequences can form the basis of a sensitive and specific, robust and informative test for the detection of cancer. A spectrum of methods is available for the identification and quantitation of methylated DNA. These include cytosine deamination PCR, semi-quantitative and quantitative methylation-specific PCR (MSP), differential methylation hybridization (DMH), restriction landmark genomic scanning (RLGS), single-nucleotide primer extension (SNuPE), pyrosequencing, and methylation microarray for large-scale genome analysis. However, MSP is a simple and sensitive method, and is the most commonly employed method for methylation analysis.

Although several hundreds of biomarkers have been found promising for cancer diagnosis, only a handful of biomarkers have been approved by the US Food and Drug Administration during the past two decades [20]. This is because most biomarkers lack sufficient sensitivity and/or specificity. To be useful, biomarkers must distinguish between people with cancer and those without. No single biomarker is likely to have 100% sensitivity and 100% specificity for a particular neoplasm. Instead, panels of biomarkers seem to be a promising alternative for the use in clinical laboratories. For example, GSTP1 methylation is detected as low as one prostate cancer cell, but not specific to prostate cancer as the GSTP1 methylation is also present in breast and renal cancers. However, two or multiple genes cohort such as GSTP1/LGALS3 or GSTP1/RARβ2/APC has been shown to be more specific and sensitive biomarkers for prostate cancer [18,19]. However, optimization of this combined assay and its validation in large scale studies are necessary before this combined assay can be considered clinically useful.

## Figures and Tables

**Figure 1:**
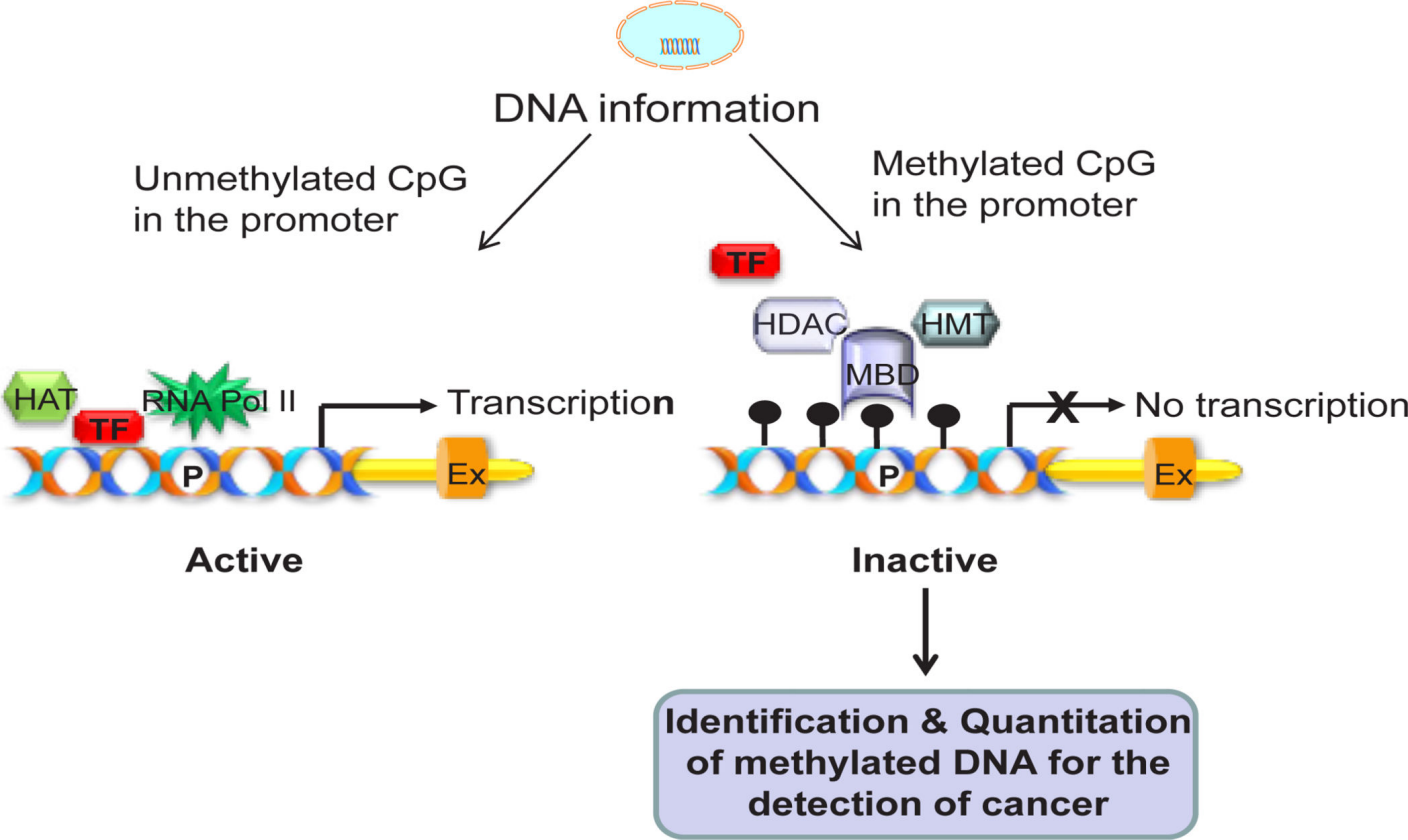
Simplified cartoon showing gene transcription by unmethylated promoter and gene silencing by the methylated promoter. In normal cells, promoter of some genes such as tumor suppressor protein, DNA repair proteins is unmethylated and accessible to binding to the transcription factors (TF) allowing transcription. But, in many cancers these genes are methylated by DNA methyltransferase 1 and therefore bound by the methyl-CpG binding proteins (MBD) and histone deacetylase (HDAC). Thus the methylated promoter is not accessible to binding to the transcription factors and inactive. In tumor tissues and biological fluids such as serum and urine, the methylated DNA is measured by various methods for the development of diagnostic and prognostic tools for the cancer. HAT indicates histone acetyltransferase; RNA pol II, RNA polymerase II; and HMT, histone methyltransferase.
